# Why dopamine replacement therapy will stay as a cornerstone in the treatment of Parkinson disease: a personal opinion

**DOI:** 10.1007/s00702-026-03205-0

**Published:** 2026-06-18

**Authors:** Heinz Reichmann

**Affiliations:** Department of Neurology, University of Technology Dresden, Fetscherstrasse 74, 01307 Dresden, Germany

**Keywords:** Parkinson disease, Dopamine, Dopamine replacement therapy, Levo-dopa, Dopamine agonists, COMT inhibitors, MAO-B inhibitors

## Abstract

In 1961 the use of levodopa was introduced into the treatment of Parkinson’s disease (PD). Since bradykinesia seems to reflect the death of dopaminergic nigrostriatal neurons, this observation makes sense. To date, levodopa in combination with a decarboxylase inhibitor is the safest and most efficacious oral treatment in PD. Over the last decades, motor fluctuations, the most important roblem associated with levodopa and due to its short plasma half life have been improved by the addition of MAOB-, COMT-inhibitors and dopamine agonists. Long-acting levodopa formulations are available to keep wearing-off low and to allow the use of 2–3 levodopa tablets per day. Rescue medication, such as oral soluble levodopa/carbidopa or sublingual apomorphine is available as is subcutaneously applied levodopa/carbidopa, a new tool in advice-guided therapy to come near to physiological continuous dopamine receptor stimulation and in this way allow persistent motor control. Most subtances used in PD treatment function via the dopaminergic system. One exception is amantadine, which shows anti-glutamatergic function. At present, modern treatment of PD is a dopamine replacement therapy and probably this will stay that way for a long time to come.

## Pathogenesis of Parkinson‘s disease

It is generally accepted that the movement disorder Parkinson‘s disease (PD) results from a loss of dopamine producing cells in the nigrostriatal system (Tretiakoff 1919). The German neuropathologist, Friedrich Jacob Heinrich Lewy, detected what is in tribute to him now called Lewy bodies (Lewy 1912). Braak and his colleagues were the first to describe the spreading of this Lewy body pathology in the brain and in the autonomic nervous system (Braak et al. 2002). A further development in the description of Lewy body distribution in the body of PD patients comes from the Borghammer Group, which indicates that there is body first and brain first pathology, which explains some features such as asymmetry, present or missing autonomic failure, dementia and much more (Horsager and Borghammer 2024). Polymeropoulos et al. (1997) were the first to detect alpha-synuclein as a major contributor to Parkinson disease and in the same year, Spillantini et al. (1997) published that Lewy bodies contain pathological alpha-synuclein. Even if this Lewy/alpha-synuclein pathology is widespread, the movement disorder with bradykinesia, resting tremor, and rigidity stems from a dopamine loss due to degeneration of dopaminergic neurons in the substantia nigra. The threshold seems to be 60% loss of dopaminergic neurones in the substantia nigra before motor symptoms occur (Fearnley and Lees 1991).

## Clinical diagnosis of Parkinson’s disease

The diagnosis of PD is established mostly by clinical means and according to the guidelines of the Movement Disorder Society (Postuma et al. 2015) and the new German guidelines on the diagnosis and treatment of Parkinson’s disease (Hoeglinger and Trenkwalder 2024). Bradykinesia has to be present and possibly rigidity and resting tremor as well. Both guidelines underline that a positive result from dopamine replacement therapy strongly supports the diagnosis of PD. The so-called dopaminergic challenge tests include the acute levodopa challenge test, acute apomorphine challenge test, and chronic levodopa therapy to assess improvement of motor symptoms (Abramsky et al. 1971; Barker et al. 1989). Recently, Kou et al. (2025) have provided a meta-analysis on publications involving these challenge tests and concluded that there is a high responsiveness in PD patients, thus, in my view this is a strong argument that addressing the dopaminergic system is the cornerstone in PD treatment.

## Treatment of Parkinson’s disease

Modern PD treatment is mostly a dopamine replacement therapy. In the history of drug development, belladonna and later anticholinergics were the first substances that improved PD symptoms, especially tremor, and did not addressing the dopaminergic system. Ordenstein (1867) reported that treatment of hypersalivation of PD patients with Atropa belladonna, which acts as an anticholinergic, resulted in improvement of resting tremor. Katzenschlager et al. (2003) gave a comprehensive overview of the use of anti-cholingergics. Although these substances have a long history, they are no longer used in developed countries except against tremor. The most important reason for this reluctance is the observation that besides many side effects related to the autonomic nervous system, anticholinergics may cause psychosis and cognitive impairment (e.g. DeSmet et al. 1982).

So far, the most important break through in PD treatment was in 1961 with the introduction of levodopa as a new medication (Birkmayer and Hornykiewicz 1961, Barbeau et al. 1962; Birkmayer and Mentasi 1967). The use of levodopa remains the gold standard for PD treatment, which makes sense since it alleviates the dopamine deficiency in the nigrostriatal system. With the addition of a decarboxylase inhibitor (patent by MSD in 1961, 1963, 1969, and 1971) the bioavailability of levodopa is increased ten-fold. In 1977, the world health organisation included levodopa in combination with carbidopa into the list of essential medications. We also use a second decarboxylase-inhibitor, i.e. benserazide.

During the last decades, we have seen several new galenic forms to the application of levodopa. A very useful tool is the soluble form of levodopa, which as a solution in water reaches the small intestine faster than the tablet or capsule form, and is thus associated with a fast and good reponse (Steiger et al. 1991). A second rescue medication, which uses levodopa-carbidopa as an oral spray, has been available for a couple of years. This formulation (CVT-301, Inbrija^®^) results in a quick and reliable improvement in motor dysfuction. Its advantage is the surpassing of the esophago-gastro-intestinal system, which is often impaired (slow movements) in PD. This dopamine replacement therapy was shown to be efficacious and safe (LeWitt et al. 2019).

The next modification was a slow-release preparation of levodopa/benserazide, which for example improves nighttime motor control (Quinn et al. 1987). This was an important breakthrough since levodopa has a very short plasma half-life time of about 2 h which is increased up to 6 h with the slow-release formulation. More recently, new levodopa formulations with longer half-life times have been developed because it was demonstrated that regular levodopa with a decarboxylase inhibitor causes pulsatile dopamine receptor stimulation and not the physiologic phasic or continuous stimulation (Obeso et al. 1994; Olanow et al. 2000). In addition to the above mentioned slow-release medication IPX066 (Rytary™) has been introduced. This type of levodopa/carbidopa has been designed to provide an intial rapid absorption of levodopa like regular levodopa formulations and thereafter provide stable levodopa concentrations to avoid peaks, which cause dyskinesia, and troughs, which cause akinesia (Mittur et al. 2017, Hauser et al. 2021). In 2024, the FDA approved another long acting carbidopa-levodopa formulation (IPX2023, Crexont^®^) which may be licensed in Europe in 2026 after approval by EMA. This capsule, due to its longer action provides good on-time and needs less frequent dosing compared to regular levodopa-carbidopa or levodopa-benserazide. It contains immediate-release granules with levodopa-carbidopa for rapid onset of action, and extended-release pellets with levodopa for long-lasting efficacy (Espay et al. 2024a, b).

As mentioned above, levodopa has one disadvantage and this is its short plasma half-life time, which is also present when a decarboxylase inhibitor is added. Luckily, there are several good options to overcome this problem. The first one is the addition of a monoamine oxidase (MAO)-B inhibitor. Rasagiline is used nowadays and has replaced selegiline. Rasagiline has shown good increase in on-time in combination with other substances (Parkinson Study Group 2005) especially levodopa. Due to its inhibition of the degradation of endogeneous (produced by the remaining dopamine neurons) and exogeneous dopamine (which is produced from applied levodopa) there is more and with respect to the short plasma half-life time longer dopamine available to stimulate dopamine receptors. This results in less motor fluctuations such as wearing-off. In a second study, which was called the LARGO study (Rascol et al. 2005) rasagiline was shown to have equal efficacy to entacapone. More recently a new MAO-B inhibitor, safinamide, has been introduced which also acts as an anti-glutamatergic drug and improves levodopa efficacy (Borgohain et al. 2014).

The second strategy to improve the short plasma half-life time of levodopa is the addition of a catechol-O-methyl transferase (COMT) inhibitor. At present, three of them are available, i.e. tolcapone, entacapone, and opicapone. Tolcapone is the only one that also inhibits the COMT in the brain, whereas entacapone and opicapone selectively inhibit the peripheral COMT in the gut so that due to the inhibition of the conversion of levodopa by COMT to 3-O-methyl dopamine more levodopa can reach the brain and thus improve the short plasma half-life time. While entacapone has to be added to each levodopa dose, opicapone is mostly used in a single dose in the evening. Entacapone’s efficacy has been demontrated in various studies (e.g. Rinne et al. 1998) and can claim to enhance the response to levodopa. More recently, the new COMT inhibitor, opicapone, was introduced which shows at least equal efficacy as entacapone without diarrhea and discoloration as seen in patients treated with entacapone (Ferreira et al. 2016; Lees et al. 2017; Reichmann et al. 2022). All studies underline the improvement in wearing off and other motor fluctuations by use of COMT inhibitors, which are modifying and improving the pharmacokinetics of levodopa (and a decarboxylase inhibitor). Thus, COMT inhibitors are only used in combination with levodopa.

A third excellent tool to overcome levodopa-induced motor complications such as wearing off is the use of dopamine agonists. Dopamine agonists are substances that directly stimulate the postsynaptic dopamine receptors. At present, dopamine agonists with a long-lasting efficacy and non-ergot derivatives are mostly used, i.e. rotigotine patch, pramipexole and ropinirole extended release preparations and piribedil. Due to their continuous dopamine receptor stimulation, they normally do not cause motor fluctuations and in combination with pre-existing levodopa allow a reduction in levodopa dose and an improvement in motor fluctuations. A combination of relatively low doses of levodopa and of a dopamine agonist is in my view an excellent option to keep the side effects of both substance classes low and to add their efficacy to a good motor response (Watts et al. 2010). This again underlines the importance of dopamine replacement therapy in PD treatment.

Most recently, an old concept on the use of D1/D5 selective dopamine agonists was rejuvenated (Rascol et al. 2001; Fernandez et al. 2026; Pahwa et al. 2026). In 2001 Rascol et al. had reported on the use of ABT-431 that preverentially addressed the D1 dopamine receptors. They could show that this compound is as efficacious as levodopa but did not create less dyskinesia than levodopa. In 2026 there were two reports on a new dopamine agonist, tavapadon, which also selectively stimulates D1 and D5 dopamine agonists and which showed benefit both in the early phase of Parkinson disease and also in advanced patients. Both studies showed superiorty versus placebo with respect of more ON time and less OFF time, but both studies failed to have an active comparator, such as levodopa or a well-established dopamine agonist.

In advanced PD, motor fluctuations, falls, mood and cognitive disorders and disturbances of the autonomic nervous system are the major contributors to a massive impairment of quality of life. Some of these symptoms, especially motor control, can be improved by invasive methods such as deep brain stimulation and by various device-assisted treatment options. The oldest method by which to apply continuous dopaminergic therapy is the use of an apomorphine pump. This was recently re-evaluated by Isaacson et al. (2025) when they used subcutaneous apomorphine application in patients with a disease duration of 10 *±* 6 years for up to one year. Good on-time was increased on average by about 3 h. The most abundant side effect was skin irritation while the patients had good motor control.

Continuous levodopa-carbidopa infusion can be applied via the gut (Olanow et al. 2014) and results in a very rewarding gain in on-time of up to 4 h compared to base line. Since the pump which delivers the intestinal gel is quite heavy, it seems to be an advantage to add entacapone to the gel, which results in a much smaller pump and the efficacy is a least equal to the levodopa-carbidopa pump (Senek et al. 2017).

An intriguing new development is the application of a pre-form of levodopa-carbidopa (foslevodopa-foscarbidopa) subcutaneously via a pumping system. The addition of a phosphate group to both levodopa and carbidopa was done to adjust the ph of the gel towards that of the human skin. The hope was to improve skin tolerability when continuously applying levodopa-carbidopa subcutaneously (Soileau et al. 2022). Efficacy was superb with a gain of about 3 h on-time compared to baseline. Skin irritation was the major problem and it is very helpful to know that treating neurologists have learnt to deal with this and to diminish the percentage of skin irritation that may lead to termination of the application. In a second study by Aldred et al. (2023), the positive response on motor fluctuations was substantiated. At week 52, on-time without troublesome dyskinesia and off-time were improved from baseline on average by nearly 4 h. In 2026, we expect another subcutaneous levodopa-carbidopa formulation (ND0612). Espay et al. (2024a, b) report on good efficacy and an additional gain of 1.72 h compared to oral levodopa-carbidopa. The most common side effect was skin irritation, which was reported affect up to 83%. These side effects range however from redness to nodules.

These examples clearly demonstrate that dopamine replacement strategies are the most rewarding and convincing treatment options. Besides influence on the dopaminergic system, we have discussed some positive properties of anticholinergics. Amantadine (anti-glutamatergic action) and adenosineA2a receptor antagonists remain to be discussed. The latter class is represented by istradefylline, which is licensed in the USA and in Japan but not in Europe. Hauser et al. (2022) provided a nice overview of the function and efficacy of istradefylline. Even this substance, which addresses the adenosine system, only works in combination with levodopa and its efficacy is much lower than that of levodopa or dopamine agonists. The same is true for another non-dopaminergic drug which is mostly used in Japan, i.e. zonisamide (Goel et al. 2021) which was shown to alleviate PD symptoms to some minor extent. Non-motor symptoms were not improved by the use of zonisamide.

Amantadine is in my view the only relevant medication in PD that does not address the dopaminergic system. A non-competitive, N-Methyl-D-Aspartate (NMDA) receptor blocker, it shows positive results in PD (Schwab et al. 1969). This substance is used in early PD in some countries and is used world-wide as an anti-dyskinetic drug (Verhagen Metman et al. 1998).

## Real world data on the use of anti-Parkinson medication

There are several reports on the real world use of anti-Parkinson drugs. In a recent report from South Korea (Kim et al. 2025), amantadine was the only non-dopaminergic substance and it was only used in 3.8% of the 18,562 patients studied. Everyone received levodopa with or without MAOB/COMT inhibitor and/or dopamine agonists. In Poland (Kasprzak et al. 2025), levodopa is also considered to be the gold standard and is the most often prescribed medication, followed by dopamine agonists. In this study, which was performed in tertiary centres, levodopa was administered above the age of 50 years in almost a 100% of PD patients. Dopamine agonists were more often used in younger patients (e.g., 70% of patients below 50 years, and in 40% of patients above 70 years). The applied dose of dopamine agonists was relatively low which illustrates caution when using dopamine agonists. Anticholinergics were negligible and amantadine was prescribed less frequently than levodopa or dopamine agonists. 50% of all patients received three or more substances, which illustrates the cautious approach of clinicians towards monotherapy.

## Non-motor symptoms

Non-motor symptoms can also be improved by the use of dopamine replacement therapy. Their responsiveness can be tested by asking patients whether a given non-motor symptom improves in the on phase after application of levodopa or a dopamine agonist (Storch et al. 2013). Since dopamine agonists partially have higher affinity to certain dopamine receptor subtypes than dopamine itself they sometimes even be superior, which may be true for pramipexole, which has excellent anti-anhedonic and anti-depressive efficacy (Lemke et al. 2006; Wei et al. 2026) related to its high affinity towards D3 dopamine receptors.

## Future treatments

Dopamine cell-based replacement therapies have seen ups and downs, as illustrated in Kayhanian and Barker (2025). They report on almost 40 years using a variety of different dopamine cell sources and transplantation approaches. For ethical, logistical and technical reasons, there are not many places where such work has been performed. The development of new human stem cell-derived dopamine cells may be a door opener. An example may be a recent study of Sawamoto et al. (2025), who used induced pluripotent stem cells to create progenitor dopaminergic cells which were bilaterally implanted in seven patients. The authors claim that allogeneic iPS-cell-derived dopaminergic progenitors survived, produced dopamine and did not form tumours, therefore suggesting safety and potential clinical benefits in Parkinson’s disease.

In spite of all these exciting developments van Wamelen et al. (2024) claim that there is still a need to develop non-dopaminergic medication since symptoms such as tremor in some instances, on-related freezing, falls, camptocormia, dystonia, unpredictable dyskinesia, cognitive dysfunction, visual disturbances, orthostatic hypotension, fatigue, apathy and pain cannot be well managed by dopamine replacement therapy.

To address these important unmet needs we may either keep focusing on the underlying cause and substantiate the lacking neurotransmitter, repair cells or address the abnormal alpha-synuclein and prohibit its spreading (Pagano et al. 224). Even if these obstacles can be overcome, dopamine replacement therapy will remain the cornerstone since it is the most efficacious medication with only minor side effects. Amongst side effects, motor fluctuation is the most abundant one and for this reason new galenic forms have been developed to improve the short comes of levodopa, i.e. its short plasma half life time. We have options for rescue therapy, improvement of wearing off and other motor complications by addition of COMT- and/or MAOB-inhibitors or dopamine agonists and at the far end we have device aided therapies with levodopa pumps. Taken together, these medications all address the dopaminergic system, the malfunction of which is causing motor symptoms in PD, and thus are the cornerstone for PD treatment at present. Since the pathophysiology of PD with a loss of dopamine production in the dopaminergic neurons of the substantia nigra will stay the same even in the future no better treatment than dopamine replacement therapy will be used. Non dopaminergic treatment has only minor effects and with the exception of amantadine which improves dyskinesia these substances are hardy used and don’t share similar efficacy with dopamine replacing therapies. Due to this dopaminergic weaponary, Parkinson’s disease is the most treatable neurodegenerative disease.

## Data Availability

No datasets were generated or analysed during the current study.
